# A Soluble Humic Substance for the Simultaneous Removal of Cadmium and Arsenic from Contaminated Soils

**DOI:** 10.3390/ijerph16244999

**Published:** 2019-12-09

**Authors:** Dongxue Bi, Guodong Yuan, Jing Wei, Liang Xiao, Lirong Feng, Fande Meng, Jie Wang

**Affiliations:** 1 Yantai Institute of Coastal Zone Research, Chinese Academy of Sciences, Yantai 264003, Shandong, China; dxbi@yic.ac.cn (D.B.); jwei@yic.ac.cn (J.W.); lxiao@yic.ac.cn (L.X.); lrfeng@yic.ac.cn (L.F.); jwang@yic.ac.cn (J.W.); 2 University of Chinese Academy of Sciences, Beijing 100049, China; 3 School of Environmental and Chemical Engineering, Zhaoqing University, Zhaoqing 526061, China; 4 College of Resource and Environment, Anhui Science and Technology University, Chuzhou 233100, China; fdmeng01@foxmail.com

**Keywords:** soil pollution, soil remediation, cadmium, arsenic, humic substance, FTIR

## Abstract

With abundant oxygen-containing functional groups, a humic substance (HS) has a high potential to remediate soils contaminated by heavy metals. Here, HS was first extracted from a leonardite and analyzed for its chemical compositions and spectroscopic characteristics. Then it was assessed for its ability as a washing agent to remove Cd and As from three types of soils (red soil, black soil, and fluvo-aquic soil) that were spiked with those contaminants (Cd: 40.5–49.1 mg/kg; As: 451–584 mg/kg). The operational washing conditions, including the pH and concentration of the HS, washing time and cycles, and liquid–soil ratio, were assessed for Cd and As removal efficiency. At pH 7, with an HS concentration (3672 mg C/L) higher than its critical micelle concentration and a liquid–soil ratio of 30, a single washing for 6–12 h removed 41.9 mg Cd/kg and 199.3 mg As/kg from red soil, 33.5 mg Cd/kg and 291.5 mg As/kg from black soil, and 30.4 mg Cd/kg and 325.5 mg As/kg from fluvo-aquic soil. The removal of Cd and As from the contaminated soils involved the complexation of Cd and As with the carboxyl and phenolic groups of HS. Outcomes from this research could be used to develop a tailor-made HS washing agent for the remediation of Cd- and As-contaminated soils with different properties.

## 1. Introduction

Cadmium (Cd) and arsenic (As) accumulation in soils is a problem in some places. A national survey in China [1], for example, revealed that Cd content in 7.0% of sampling sites exceeded the national standard; for As content it was 2.7%. Because of their toxic and carcinogenic nature, Cd and As in the soil poses a threat to environmental quality, food safety, and human health. It is highly desirable to remove Cd and As from soil, thus permanently eliminating the threat. 

Various physical, chemical, and biological methods and techniques have been investigated for Cd or As removal from soil [2,3,4]. Among them, soil washing is considered to be one of the most suitable techniques for removing heavy metals from soil [5,6]. The selection of washing agents is critical to the success of soil washing technology. Factors influencing the applicability include technical reliability, costs, and side effects [7]. Washing agents can be classified into five categories: (1) inorganic acids, (2) chelating agents, (3) surfactants, (4) simple organic acids, and (5) salts (e.g., FeCl_3_ and CaCl_2_) [8,9,10,11,12]. While inorganic acids and synthetic organic chelators have a high efficiency in removing heavy metals, they produce a range of adverse effects on the physical, chemical, and biological properties of soil, from reduced water holding capacity and loss of essential nutrients to decreased enzyme activity [13,14,15,16]. Because clay minerals in soil could readily adsorb simple organic acids, a high dose of organic acids would be required to produce a noticeable washing effect. Salt like FeCl_3_ could result in soil acidification. Besides the above-inherited disadvantages of washing agents, the complexity of soil environment adds extra difficulties to the use of some washing agents. The removal of Cd and As, two contaminants with opposite chemical behaviors, is even harder to achieve. Ethylenediaminetetraacetic acid disodium salt (Na_2_EDTA), for example, is useful for the removal of cations, but weak for removing anions [17]. In contrast, organic acids and some inorganic acids extracted many more anions than cations from the soil, sediment, and mine waste [18]. For the synergetic effect, Wei et al. [6] used Na_2_EDTA, oxalic acid, and phosphoric acid in sequence to wash Cd- and As-contaminated soil, at the expense of adding operational complexity and cost.

This research aimed to extend the use of HS as a washing agent [5] to the simultaneous removal of cadmium and arsenic from contaminated soil. To this end, a water-soluble HS, easily obtained from a leonardite, was used for the assessment of pH and other washing conditions with regard to its efficiency in removing Cd and As from spiked soils; in addition, spectroscopic changes in the functional groups of the HS during the washing were analyzed to reveal the mechanisms involved in the removal of Cd and As from the soils. Besides being rich in carboxyl and phenolic–hydroxyl groups [19], and thus capable of removing Cd and As from soil, HS has a number of advantages over other washing agents, in that it is cheaply available from coal, peat, and sludge [20], and has beneficial effects on soil physical, chemical, and biological properties [21]. 

## 2. Materials and Methods 

### 2.1. Soil Samples, Spiking Treatment, and Laboratory Analysis

Three soil samples were obtained from the surface layer (0–20 cm) of soils to represent three major soil types in China, including a red soil (Ferralsols) at Yingtan Station in the Chinese Ecosystem Research Network (28°12′ N, 116°55′ E), Jiangxi Province; a black soil (Phaeozems) from Jilin Province (43°30′ N,124°48′ E); and a fluvo-aquic soil (Fluvisols) from Tianjin City (39°23′ N,117°51′ E). The soil samples were air-dried, gently crushed, and sieved through a 2 mm nylon mesh for use. 

The soil samples were spiked with a solution of Cd(NO_3_)_2_·4H_2_O and Na_3_AsO_4_·12H_2_O, mixed well, moisturized at 15% water content, and aged in the dark for 5 months. The particle size distribution of the soil samples was determined by a particle size analyzer (Mastersizer 2000, United Kingdom). The pH was determined using a distilled water (1:5 ratio, *w*/*v*) by a pH meter (Mettler Toledo FiveEasy Plus, Switzerland). The cation exchange capacity (CEC) and total Cd and As concentrations of the soil samples were analyzed following the standard methods, as described [22]. Briefly, neutral NH_4_Ac was used for CEC measurement of red soil, whereas NaAc (pH 8.2) was used for the determination of the CEC of the black and fluvo-aquic soils. Soil organic carbon (SOC) content was determined by an elemental analyzer (Vario Micro cube, Elementar, Germany). For the fluvo-aquic and black soils, pretreatment with 1 mol/L hydrochloric acid was conducted to remove calcium carbonates before SOC determination. Spiked soil samples were digested with HClO_4_-HNO_3_-HF at a 3:1:1 ratio (*v*:*v*:*v*) and analyzed by Inductively coupled plasma mass spectrometry (ICP-MS, PerkinElmer Elan DRC II, Waltham, MA, United States) for Cd and As concentrations. 

### 2.2. Humic Substance and Its Analysis 

A leonardite from the Xinjiang Uyghur Autonomous Region was used to extract an HS. Briefly, a leonardite powder was first added to a centrifuge tube with 0.1 M KOH solution at a 1:40 (g:mL) ratio. The tube was then shaken for 2 h at 298 K in a thermostatic oscillator, and centrifuged at 3000 r/min for 10 min. Finally, the supernatant was collected and oven-dried at 333 K to obtain HS for the analyses of its (1) elemental compositions using an element analyzer; (2) pH and total heavy metal concentrations, by the methods mentioned above; (3) contents of carboxyl and phenolic groups by the method of the International Humic Substances Society [23]; (4) critical micelle concentration (CMC), by plotting the relationship between surface tension and HS concentrations [24]; and (5) surface functional groups by Fourier transform infrared spectroscopy (FTIR; Thermo Fisher Nicolet iS5, Waltham, MA, USA) at 4 cm^−1^ resolution for the range of 4000–600 cm^−1^. The organic carbon content in the HS washing liquid was determined by a total organic carbon analyzer (Shimadzu TOC-VCPH, Kyoto, Japan).

### 2.3. Soil Washing Experiment

Batch experiments were conducted in triplicate at room temperature, in order to evaluate the effect of washing conditions on the removal of Cd and As by HS. The HS-deionized water suspension was added into polypropylene centrifugal tubes that contained Cd- and As-spiked soils, and the tubes were placed in a thermostatic oscillator at 300 r/min. The variables for the evaluation included solution pH (3.0, 5.0, 7.0, 9.0, 10.6), the solution/soil ratio (5, 10, 20, 30, 40 mL/g), HS concentration (0, 367, 1469, 2571, 3672, and 5508 mg C/L), washing time (0, 0.5, 1.5, 3.0, 6.0, 12, 24 h), and washing cycles (1, 2, 3). At the end of the shaking, the tubes were centrifuged at 3000 r/min for 10 min. The supernatant was freeze-dried for FTIR analysis, and soil residue in the tubes was digested by HClO_4_-HNO_3_-HF at a 3:1:1 ratio (*v*:*v*:*v*), as above, to determine Cd and As in the soil residue. 

### 2.4. Kinetics of Cd and As Removal by Humic Substance

The following models were used to describe the desorption of Cd and As from the spiked soils and assess the effect of HS on Cd and As removal. 

 Pseudo-first-order equation: 
$q_{t}=q_{1}(1-e^{\left(-k_{1}t\right)})$
(1) Pseudo-second-order equation: 
$q_{t}=\frac{q_{2}^{2}k_{2}t}{1+q_{2}k_{2}t}$
(2) Elovich: 
$q_{t}=\frac{1}{\beta }\ln(\alpha \beta )+\frac{1}{\beta }\ln(t)$
(3) Parabolic diffusion: 
$q_{t}=a+k_{p}t^{1/2}$
(4) Power function: 
$\ln q_{t}=\ln b+k_{f}(\ln t)$
(5)

Here, $q_{1}$ or $q_{2}$ is the amount of Cd or As adsorbed by HS at equilibrium (mg/kg), respectively; $q_{t}$ is the amount of adsorbed As or Cd at time *t* (mg/kg); $k_{1}$ and $k_{2}$ are the equilibrium rate constants; α is the initial release rate (mg/(kg·h)); β is the release constant (mg/(kg·h)); $k_{p}$ is the diffusion rate constant (mg/kg)^−0.5^; $k_{f}$ is the rate coefficient value (mg/(kg·h)); and *a* and *b* are constants.

## 3. Results and Discussion

### 3.1. The Basic Properties of Spiked Soils and Humic Substances

As shown in Table 1, the spiked soils were similar in soil texture (silty loam), but different in pH. The black soil had the highest CEC, because of its highest organic carbon content. The content of Cd and As in the spiked soils exceeded the control values listed in *Risk Management and Control Standards for Soil Pollution* in China (GB336600-2018).

HS had a large C/N ratio of 78.65 (Table 2), suggesting it would be difficult to be decomposed if it is added to the soil [25]. The small H/C ratio (0.07) indicates high aromaticity. The O/C ratio (0.58) is related to the number of oxygen-containing functional groups: the larger the ratio is, the more oxygen-containing functional groups there are, which is consistent with the content of carboxyl and phenolic functional groups. The CMC of HS (1575 mg C/L) is lower than those of humic acid (1890 mg C/L) and fulvic acid (3150 mg C/L) used in [5], suggesting that the HS of this study is a more powerful washing agent.

### 3.2. Effect of pH on the Removal of Cd and As 

**Figure 1:**
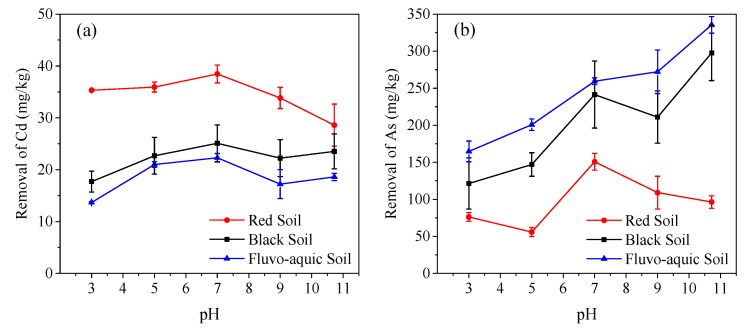
Effect of pH on Cd (**a**) and As (**b**) removal from spiked soils by a single washing with a humic substance (HS) (soil: solution ratio of 1 g:20 mL).

A 24 h washing of the Cd- and As-spiked soils with HS at the same concentration of 3672.4 mg C/L but different pH values showed that the amount Cd removal at pH 7 was slightly more than at acidic or alkaline pHs (Figure 1). An acidic pH favored the protonation of functional groups (e.g., carboxyl), reduced the repulsion among HS molecules, and formed a crimped compact structure [26], thus lowering its ability to remove Cd from soil particles. At alkaline pH, the competition between the formation of Cd(OH)_2_ and the Cd–HS complex reduced the amount of Cd removed from the spiked soils. HS removed more Cd from red soil than from black and fluvo-aquic soils, which could be attributed to the lower organic carbon content, and thus the lower ability of red soil to bind Cd than the other two soils.

The effect of pH on As removal was more complicated than Cd. HS removed less As from red soil than from black and fluvo-aquic soils. In red soil, As compounds (e.g., H_2_AsO_4_^−^ and HAsO_4_^2−^) tend to be immobilized by the Fe(Al, Mn)-(hydr)oxides. At an acidic pH, the ability of compact and protonated HS to bind with Fe(Al, Mn)-(hydr)oxides was low, and so was its ability to replace As compounds for their release into solution. With the increase of pH to neutral, the carboxyl groups of HS became more deprotonated, their ability to bind with Fe(Al, Mn)-(hydr)oxides increased, and As release was enhanced. At high pH levels, Fe(Mn) would be precipitated as hydroxides, in order to adsorb negatively charged As compounds again. In black and fluvo-aquic soils, as pH increased, the surface of soil particles became more negatively charged and repulsive to As compounds (e.g., AsO_4_^3−^), thereby promoting the desorption of As [27]. Though Ca^2+^ could co-precipitate with As under alkaline conditions [28], the strong ability of HS to bind Ca^2+^ in black and fluvo-aquic soils suppressed the co-precipitation. 

To balance the Cd and As removal efficiency with the destructive effect of very acidic or alkaline conditions on soil physical, chemical, and biological properties, a near-neutral pH is recommended for HS. From the operational point of view, HS liquid with a near-neutral pH is also easy and safe to handle. 

### 3.3. Effect of Liquid–Solid Ratio on the Removal of Cd and As

**Figure 2:**
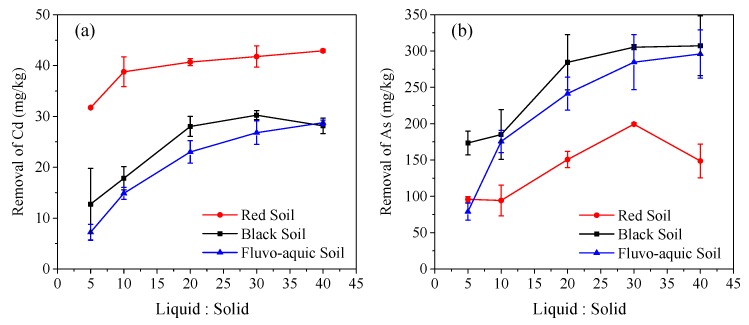
Effect of liquid–solid ratio on the removal of Cd (**a**) and As (**b**) by a single washing.

HS at the concentration of 3672.4 mg C/L and pH 7 was used to assess the effect of the HS liquid–soil ratio on Cd and As removal by single washing for 24 h (Figure 2). A high ratio provides more HS to bind Cd or weaken the association of As with Fe (Al, Mn)-hydroxides, thus promoting the release of Cd and As from the spiked soils. At the ratio of 40:1, 90.3% of the Cd in the red soil was removed. The corresponding value was lower in fluvo-aquic soil (71.0%) and black soil (61.6%), reflecting the ability of soil organic matter to adsorb Cd in the following order: red soil < fluvo-aquic soil < black soil. The removal percentage of As was up to 52.6% in the black soil, up to 52.2% in the fluvo-aquic soil, and up to 44.2% in the red soil. HS provides adsorptive sites for Ca^2+^ in fluvo-aquic and black soils, weakened the binding of As with Ca, and enhanced As release from the soils. 

### 3.4. Effect of Humic Substance Concentration on the Removal of Cd and As

**Figure 3:**
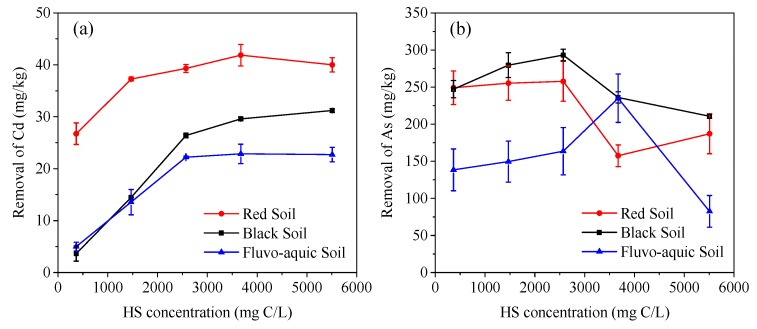
Effect of HS concentration on the removal of Cd (**a**) and As (**b**) by a single washing.

For a single washing of 24 h at the HS liquid–soil ratio of 20 and pH 7, the amount of Cd removed from the spiked soils increased with HS concentration (Figure 3). This effect is similar to that of increasing the HS liquid–soil ratio, as discussed in Section 3.3. The relationship between the HS concentration and As removal is more complicated. 

At a concentration lower than critical micelle concentration (CMC, 1575 mg C/L), HS existed in a molecular state [29]. When the concentration was higher than CMC, HS molecules turned to micelles, resulting in a decreased availability to react with Fe(Al, Mn)-hydroxides and Ca^2+^ to release As from the soils. Because soil minerals can adsorb HS [21] and reduce its concentration in an aqueous system, a concentration higher than CMC would be required for HS to form micelles in a soil–water suspension. Thus, the maximal As removal percentage occurred at HS concentrations higher than CMC (Figure 3).

### 3.5. Effect of Humic Substance Washing Cycles on Cd and As Removal 

**Figure 4:**
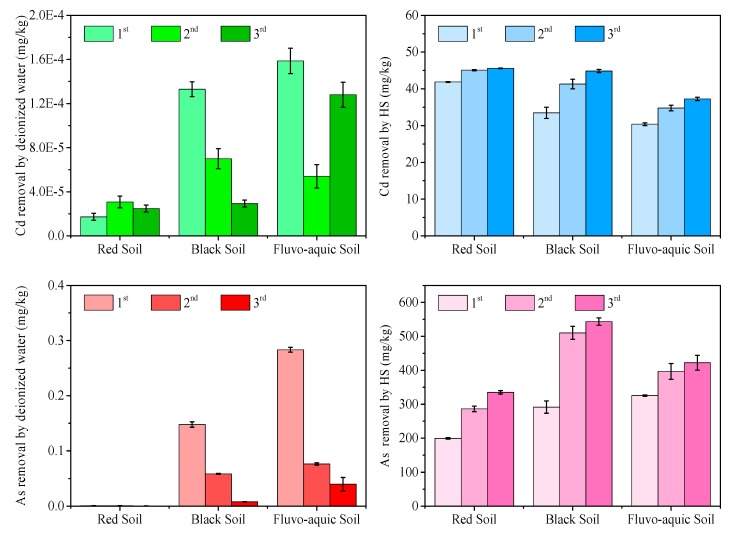
Effect of HS washing cycles on Cd and As removal.

Figure 4 shows the effect of washing cycles on Cd and As removal at an HS concentration of 3672.4 mg C/L and pH 7. Deionized water (as the blank) removed a negligible amount of Cd and As. An increase in washing cycles from 1 to 3 enhanced the removal of both Cd and As. However, the enhancement was more significant for As than Cd, and varied among soils. Washing cycles had the most significant effect on black soil (Cd removal increase by 23.4% on the second washing and 8.6% on the third washing), suggesting that the high soil organic carbon content in black soil played a role by protecting Cd in soil from being removed by extraneous HS. Repeated washing had the greatest effect on As removal from black soil: 93.0% of As was removed on the third washing, versus 74.5% from the fluvo-aquic soil and 74.3% from the red soil. As organic matter competed with As for adsorption onto minerals in soil [30], repeated washing by HS helped the release of As from soil.

### 3.6. FTIR Analysis of Humic Substance Before and After Washing Contaminated Soils

**Figure 5:**
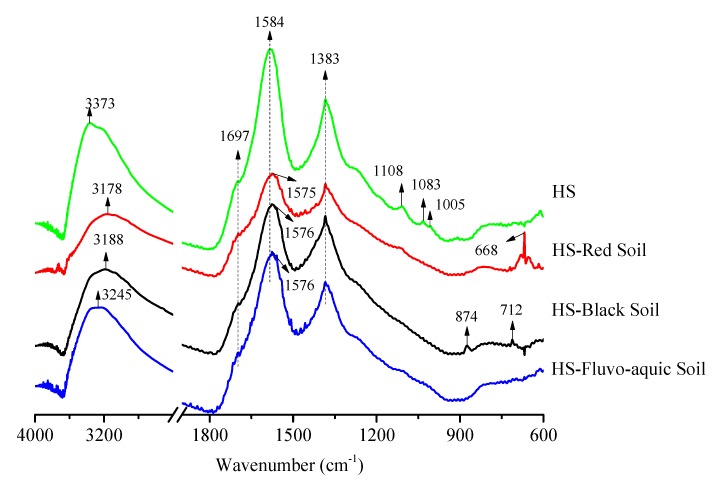
FTIR of HS before and after washing soils.

The FTIR spectra of the HS itself and the supernatant (freeze-dried) from soil washing are shown in Figure 5. In agreement with the high carboxyl and phenolic hydroxyl groups determined by the titration method of IHSS (International Humic Substances Society), the HS contained a large number of oxygen-containing functional groups, with the ability to donate electron pairs and to bind Cd and As [31]. At 3373 cm^−1^ was –OH and part of the N–H stretching vibration [32]. At about 1700 cm^−1^ was the stretching vibration of C=O [33,34]. At 1584 cm^−1^ was the stretching vibration ($v_{a}$, asymmetric stretching) of C=C in COO^−^, and the peak at 1383 cm^−1^ was attributed to the vibration ($v_{s}$, symmetric stretching) of COO^−^ [33,35]. At 1300–1000 cm^−1^ was the C–O stretching vibration of esters and anhydrides [36]. The peak at 1108 cm^−1^ was due to the stretching of C–O [37]. The peak at 1083 cm^−1^ was in the region of the C–O–C bond vibration [38]. At 1005 cm^−1^ was a C–O stretch of carbohydrate and polysaccharide [39]. 

After washing the spiked soils, the HS showed some changes. The –OH peaks at 3373 cm^−1^ shifted to 3178, 3188, and 3245 cm^−1^, and the absorption peaks weakened obviously, suggesting the adsorption of Cd and As on the –OH groups. The arsenate center has a form charge of +V. The addition of a phenolate at the electrophilic center would cause protonation and water release, resulting in the bonding of arsenate with HS [40]. Meanwhile, the characteristic peak (1584 and 1383 cm^−1^) intensities of the carboxyl group decreased gradually, which may be due to the deprotonation of the carboxyl functional group [41]. This group can combine with Cd to form carboxylates. The reduction of Δ = $v_{a}$ − $v_{s}$ was attributed to chelation (metal bound to two O atoms) or bridging recombination (one metal bound to each O atom) [42]. After washing the red soil and black soil, three peaks (874, 712, and 618 cm^−1^) appeared in the fingerprint area of HS, mainly the stretching vibration of the C–H or C–O–C bond of the aromatic ring [43]. This may be due to the formation of a new chemical bond after Cd and As combined with the aromatic ring of the HS molecules. The spectroscopic results indicated that carboxyl and hydroxyl groups on the surface of HS were the primary binding sites for Cd and As, and the mechanisms involved chelation and bridging recombination. 

### 3.7. Kinetics of Cd and As Removal by Humic Substance

**Figure 6:**
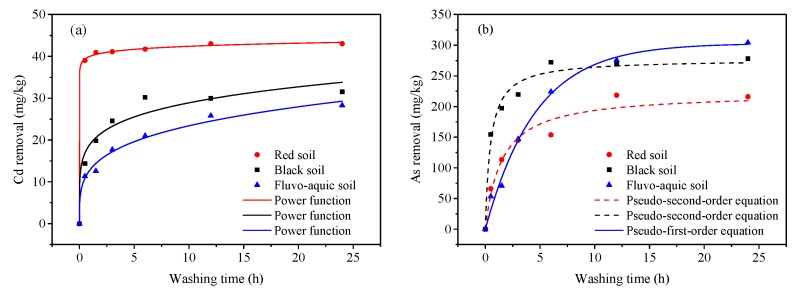
Kinetics of Cd (**a**) and As (**b**) desorption from contaminated soils.

Kinetic models have been used to describe the rate of heavy metal desorption from the soil [44,45]. Here, we used five dynamic models to compare the desorption of Cd and As from the soil (Table 3). The high *R*^2^ values indicate that the power function model described the Cd desorption process well in the spiked soils, whereas As release from the soils was better described by the Pseudo-first-order and Pseudo-second-order kinetic models. Because the desorption rate can be judged by the kinetic constant k [46,47], desorption of As was the fastest from the black soil, and Cd desorption was fastest from the red soil (Table 3 and Figure 6). 

## 4. Conclusions

Cd- and As-spiked soils can be effectively decontaminated by using the humic substance as a washing agent and tailoring washing conditions (pH, HS concentration, washing solution–soil ratio, washing duration, and washing cycles) to soil properties. A single washing of red soil for 12 h at pH 7, liquid–solid ratio of 30, and HS concentration of 3672 mg C/L removed 88.1% of the Cd and 44.2% of the As in the soil. For black soil, a single washing (6 h) removed 68.2% of Cd and 49.9% of As. A single washing of fluvo-aquic soil (12 h) removed 75.1% of Cd and 57.4% of As. Repeated washing further improved the removal rate. A near-neutral pH of HS was suitable for soil washing to achieve a reasonable removal rate and minimize the destructive effect on soil. Its high efficiency in removing Cd and As from soils and its low metal contents (Cd: 0.09, Pb: 5.32, Cu: 8.72, Cr: 6.60, As: 3.69, Ni: 8.39, ZnL 20.36 mg/kg) determine that the HS could be safely used for the remediation of Cd- and As-contaminated soil. Field trial verification of this washing method could be performed in paddy soil, for example, by combing HS addition with routine farming practice (ploughing and drainage) for the removal of Cd and As from contaminated soils. The mechanisms involved in Cd and As removal from soil by HS could include outer- and inner-sphere complexation of Cd with HS, ternary humic acid-cation-arsenic complexation, and the binding of As with phenolic groups of HS.

## Figures and Tables

**Table 1:** Soil properties and metal concentrations in the spiked soils.

Parameters	Unit	Red Soil	Black Soil	Fluvo-Aquic Soil
Sand content	%	31.53 ± 0.09	32.79 ± 0.05	36.36 ± 0.05
Silt content	%	55.77 ± 0.15	55.41 ± 0.14	51.56 ± 0.14
Clay content	%	12.71 ± 1.04	11.80 ± 0.59	12.08 ± 2.13
Texture ^1^	-	Silty loam	Silty loam	Silty loam
CEC	Cmol (+)/kg	11.01 ± 0.13	26.05 ± 0.21	18.41 ± 0.17
Soil organic carbon	g/kg	2.72 ± 0.03	16.43 ± 0.14	10.52 ± 0.07
pH (H_2_O)	-	5.68	7.89	8.20
Total Cd	mg/kg	47.50 ± 0.55	49.10 ± 0.93	40.47 ± 0.93
Total As	mg/kg	450.86 ± 10.12	584.25 ± 8.56	566.88 ± 10.47

^1^ International soil texture classification standards.

**Table 2:** Basic properties of the humic substance.

Parameters	C	H	O	N	Ash Content	pH	CMC ^1^	Phenolic-OH	-COOH	Total Ca	Total As	Total Cd
Unit	%					-	mg C/L	mol/kg		mg/g	mg/kg	
Humic substance	40.90	2.82	23.71	0.52	31.78	10.72	1575.46	2.84	7.55	12.13	3.69	0.09

^1^ Critical micelle concentration.

**Table 3:** Kinetics model parameters of Cd and As desorption from soils.

Models	Parameters	Metal	Red Soil	Black Soil	Fluvo-Aquic Soil
Pseudo-first-order equation	*q*_1_	Cd	41.960	29.820	25.710
	*k*_1_		5.294	0.846	0.457
	*R* ^2^		0.997	0.952	0.882
	*q*_1_	As	203.320	258.650	303.070
	*k*_1_		0.461	1.342	0.217
	*R* ^2^		0.918	0.931	0.990
Pseudo-second-order equation	*q*_2_	Cd	41.48	25.05	19.45
	*k*_2_		0.847	0.145	0.144
	*R* ^2^		0.996	0.842	0.737
	*q*_2_	As	222.340	276.780	321.030
	*k*_2_		0.003	0.008	0.001
	*R* ^2^		0.968	0.983	0.972
Elovich	*α*	Cd	5260.98	252.05	70.39
	*β*		0.222	0.214	0.209
	*R* ^2^		0.804	0.913	0.950
	*α*	As	424.160	7861.220	214.630
	*β*		0.024	0.029	0.014
	*R* ^2^		0.944	0.903	0.939
Parabolic diffusion	*a*	Cd	23.870	9.620	5.500
	*k_p_*		5.650	5.740	5.400
	*R* ^2^		0.240	0.674	0.864
	*a*	As	40.530	99.760	14.210
	*k_p_*		43.490	47.820	67.370
	*R* ^2^		0.837	0.600	0.919
Power function	*b*	Cd	40.040	19.120	12.980
	*k_f_*		0.025	0.179	0.256
	*R* ^2^		0.999	0.956	0.986
	*b*	As	99.930	187.340	91.210
	*k_f_*		0.266	0.143	0.405
	*R* ^2^		0.956	0.971	0.939
